# Screen-Printing Fabrication and Characterization of Stretchable Electronics

**DOI:** 10.1038/srep25784

**Published:** 2016-05-13

**Authors:** Jari Suikkola, Toni Björninen, Mahmoud Mosallaei, Timo Kankkunen, Pekka Iso-Ketola, Leena Ukkonen, Jukka Vanhala, Matti Mäntysalo

**Affiliations:** 1 Tampere University of Technology, Department of Electronics and Communications Engineering, Tampere, Korkeakoulunkatu 3, FI33720, Finland

## Abstract

This article focuses on the fabrication and characterization of stretchable interconnects for wearable electronics applications. Interconnects were screen-printed with a stretchable silver-polymer composite ink on 50-μm thick thermoplastic polyurethane. The initial sheet resistances of the manufactured interconnects were an average of 36.2 mΩ/◽, and half the manufactured samples withstood single strains of up to 74%. The strain proportionality of resistance is discussed, and a regression model is introduced. Cycling strain increased resistance. However, the resistances here were almost fully reversible, and this recovery was time-dependent. Normalized resistances to 10%, 15%, and 20% cyclic strains stabilized at 1.3, 1.4, and 1.7. We also tested the validity of our model for radio-frequency applications through characterization of a stretchable radio-frequency identification tag.

Over the past few years, numerous applications of wearable electronics have emerged in the consumer market, applications such as smart watches^1^, which extend the functionality and potential of traditional watches, and head-mounted displays^2^, which provide augmented reality vision. In addition, wearables are making an impact in the sports industry with wrist-worn activity trackers helping users to measure their physical activities gaining popularity. In the healthcare industry, similar wearables have been studied for unobtrusive monitoring of vital signs, such as blood pressure and electrocardiography (ECG)^3^,^4^. These applications may well revolutionize the healthcare industry, which is in urgent need to monitor patients remotely^5^ to improve the quality of patient life and to promote efficient use of hospital facilities and services. In addition, data shows that diseases can be diagnosed remotely and patients invited for examination based on set alarm limits of various vital signs. In an effort to achieve wireless and battery-free sensors, passive radio-frequency identification (RFID) tags equipped with antennas designed to function as sensing elements have been found a compelling approach^6^. In this area, demonstrations related to stretchable electronics include uni- and bi-axial strain gauge tags^7^,^8^ comprising antennas built from stretchable electro-textiles. Concurrently, RFID-inspired wireless sensing empowered by enhanced ambient energy harvesting capabilities and textile-integration of wearable electronics have emerged as major research themes^9^,^10^. Concurrently, RFID-inspired wireless sensing empowered by enhanced ambient energy harvesting capabilities and textile-integration of wearable electronics have emerged as major research themes^9^,^10^.

However, wearable electronics applications are facing numerous challenges. One is the unobtrusiveness of the device. A key technology to minimize obtrusiveness is stretchable electronics. In contrast to conventional electronics manufactured on rigid circuit boards, such as silicon, or flexible electronics manufactured on flexible circuit boards, such as polyimide (PI), stretchable electronics are manufactured on ultra-thin elastomer substrates, such as polyurethane (PU) or polydimethylsiloxane (PDMS)^11^. Stretchable electronics can be used, e.g., in applications where their functionality is embedded in human skin^12^,^13^. In addition, stretchable electronics can be integrated into textiles to add functionality to clothing^14^,^15^. In skin-affixed and textile-integrated applications, about 15-to−20% strains occur throughout the life cycle of the product^16^. This sets the requirement for strains that stretchable interconnects should be able to withstand.

Conductive traces can be embedded in elastomer substrates in various ways. Conductive patterns have been fabricated, e.g., by etching^17^, screen-printing and stencil printing^18^, and inkjet printing^19^. Intrinsically stretchable materials, such as polymers (e.g., PEDOT:PSS^20^) and CNT compounds^21^, generally permit high elongations but suffer from high resistance, whereas metal interconnects are less resistant yet usually not suitable for high elongation. Most techniques (e.g., wavy ribbons^22^ and popup structures^23^) involve pre-stretching the substrate to produce buckling in the attached conductor. An alternative way is to plane-pattern conductors into sinusoidal, zig-zag, horseshoe, or mesh-shaped patterns^11^. For example, a proper geometry makes metal interconnects highly reliable (>100 000 cycles) even at 40% elongation^24^. In addition to above mentioned solutions based on stiff metals, liquid metal alloys have been studied as potential stretchable conductive material^25^,^26^,^27^. Intrinsically stretchable conductor materials simplify design and printing technologies enables large area cost effective manufacturing. In this paper, we characterize an intrinsically stretchable conductor based on silver flakes in polymer matrix.

In this study, three lots, each of ten samples, were manufactured by screen-printing. A strain test pattern was used for the stretchable interconnects. Samples were screen-printed on 50-μm thick thermoplastic polyurethane (TPU), and conductors were made of a commercially available, flexible and conductive silver-flake based ink. TPU was chosen as substrate mainly for its thermoformability, which may be exploited to integrate stretchable electronics into textiles. TPU also has a high abrasion resistance, which makes a textile-integrated application comfortable to wear. Because of its high surface energy, TPU provides significantly better adhesion between conductor material and substrate than, e.g., PDMS without any additional surface treatment^7^. After the stretchable interconnects were fabricated, they were characterized by first evaluating their initial electrical properties and then by evaluating their electromechanical performance. Their initial electrical properties were evaluated by first measuring them for 2-point resistance. Then, for a general estimation of their initial electrical properties, sheet resistances were calculated from measured resistances with the dimensions of the desired pattern. Electromechanical performance was evaluated with a single, uniaxial stretch of each sample to a point where they lost conductivity. Based on this data, the ratio of resistance to strain was determined and the amount of strain required to break the samples. Because, as mentioned above, this proportionality is non-linear, a cubic regression model is proposed in this article. Cyclic strain tests of up to 1000 cycles with 10%, 15%, and 20% strains were performed. Results showed that resistances increase as a function of cycle count. However, they recovered almost fully after the strain was removed. Finally, a stretchable antenna for a radio-frequency application fabricated by screen-printing was demonstrated, and its performance with high elongation was measured. The simulations and experiments produced an excellent match.

## Results and Discussion

### Screen-Printing Fabrication of Stretchable Interconnects

**Figure 1:**
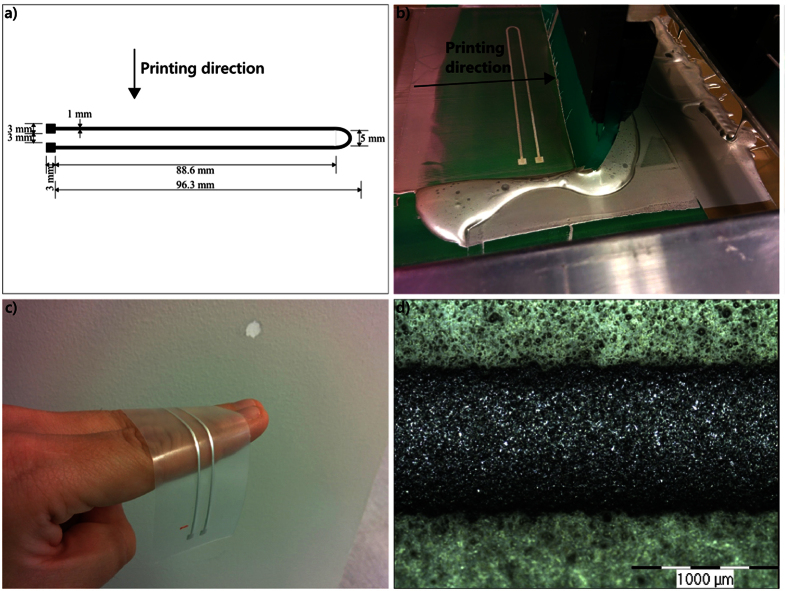
Screen-printed strain test patterns. (**a**) The pattern designed for strain testing. (**b**) Screen-printing of strain test patterns. (**c**) Flexibility of stretchable interconnects fabricated on 50-μm thick TPU substrate. (**d**) Optical microscope image of the trace of a printed stretchable interconnect.

In our study, one pattern was used in all electrical and electromechanical tests, namely a single-line structure, which is curved so that the pads are located at the same end. This pattern was chosen with strain testing in mind. In the tests, the side with the pads attached was held static, while the other end was moving. Connectors to measure resistance could then be attached to one end to avoid unnecessary measurement noise (strain test pattern shown in Fig. 1a). The line had a total length of 188.9 mm and a defined trace width is 1.0 mm. Stretchable interconnects were manufactured by screen-printing. Traces were printed on a 50-μm thick thermoplastic polyurethane (TPU) substrate, and three lots, each of ten samples, were manufactured. The total number of samples was 30. Owing to the softness of the thin TPU, substrate sheets were tightly attached to 2.0-mm thick aluminum plates with adhesive tape. The material printed on the substrate to form conductive traces was a highly conductive and highly flexible silver flakes-based ink, provided by ECM (a printed image shown in Fig. 1b). After the pattern was printed on the substrate, the traces were cured at 125 °C for half an hour. Final composition of interconnect is silver flakes in polymer matrix. Finally, the substrate was cut to a size of 38 × 140 mm^2^. The resulting stretchable interconnects were ultra-thin and ultra-flexible, as may be observed from Fig. 1c. When ready, the samples were reviewed with an optical microscope before further testing (a microscope image shown in Fig. 1d) for possible faults affecting the results of initial electrical or electromechanical measurements. The samples were also reviewed for variations in trace width. The mean value of their trace width was 1020 μm. In addition, they were reviewed for trace thickness with a Dektak 150 profilometer. The mean value of their trace thickness was 6.4 μm.

### Electrical Properties of Stretchable Interconnects

**Figure 2:**
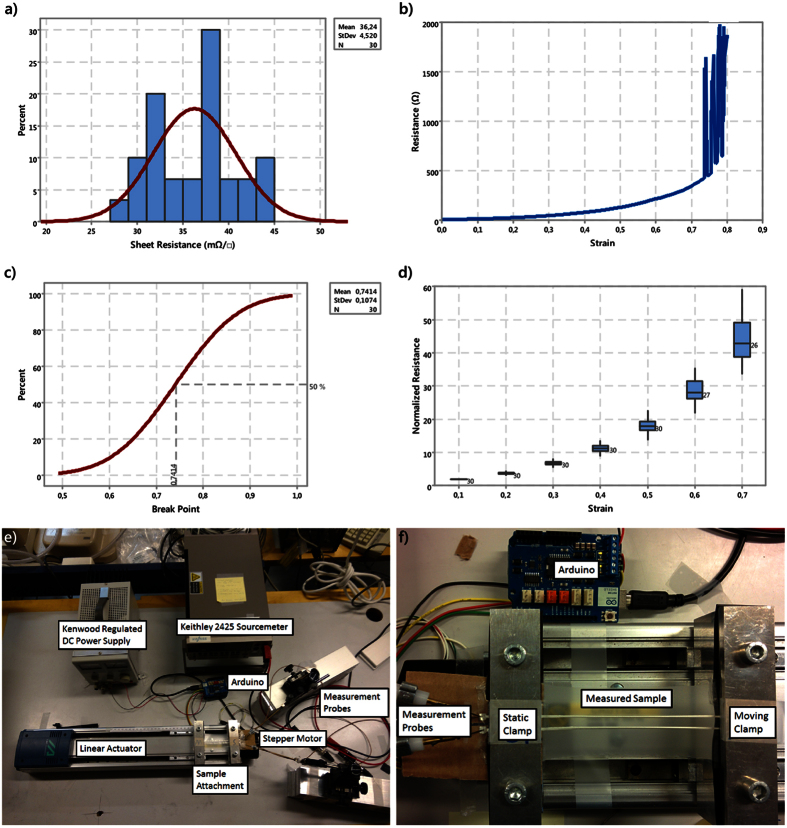
Review of the initial electrical and electromechanical properties of fabricated stretchable interconnects. (**a**) Histogram of the initial sheet resistances of the fabricated stretchable interconnects. (**b**) Proportionality of strain of an example resistance for one stretchable interconnect. (**c**) Cumulative distribution function of break points of the measured 30 stretchable interconnects. Half the samples broke before 74% strain. (**d**) Boxplot of normalized resistance as a function of the strain performance of the 30 samples. The number of still conductive samples is shown next to each box. (**e**) The whole strain test setup. (**f**) Sample attachment area in the strain test setup.

The material combination was measured for its electrical properties from the strain test pattern with a 2-point resistance measurement. Sheet resistances were calculated from the measurement data to represent the initial electrical properties of the material combination (sheet resistances of the 30 samples are shown in Fig. 2a). The figure shows that the 30 samples have a mean sheet resistance of 36.2 mΩ/◽ and a standard deviation of 4.5 mΩ/◽. For the measured average trace thickness of 6.4 μm, this corresponds to a resistivity of 2.32·10^−7^ Ωm and a conductivity of 4.31·10^6^ S/m, which is about ten times less than the conductivity of bulk silver. This surpasses the initial electrical performances of the most stretchable interconnect materials and strategy combinations reported by Yao and Zhu^28^ and is a sufficiently high conductivity for interconnects in electronics applications and even in antennas^29^,^30^.

### Electromechanical Performance of the Stretchable Interconnects

Stretchable interconnects were further characterized by stretching them uniaxially while continuously measuring their resistance. The interconnects were stretched only once, up to a strain when they lost conductivity. This is the normal procedure to characterize stretchable conductive materials for comparable results. Figure 2b shows a typical example of resistance as a function of strain for one sample and that resistance grows non-linearly in relation to strain. The point where resistance starts abruptly to increase is defined as the break point of the sample. In this article, break point is defined as the first point in strain that satisfies the condition


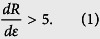


Here, R is the electrical resistance of the stretchable interconnect, and ε is the strain. The threshold value of 5 for the derivative was found by trial and error when the strain test data was examined. For example, the break point for the sample in Fig. 2b is 73% strain. A cumulative distribution function for the break points of the measured samples is shown in Fig. 2c. Based on these tests, half the samples broke at 74% strain. Figure 2d shows normalized resistance as a function of strain for the whole population. Normalization was done by dividing the resistances with the resistance value of zero strain. We can observe that the variance and range of the samples starts gradually to increase when strain increases.

The test setup for resistance as a function of strain was designed and implemented for this study. The sample attachment area in the setup is shown in Fig. 2e and the whole setup in Fig. 2f. The test setup is described in detail in the Methods below.

As mentioned in the introduction, the target strain that the stretchable interconnects manufactured in this study were to withstand was 15–20%. This was achieved in a simple strain test and verified in cycling strain test. When the normalized stretch performance of these interconnects is compared with those presented by Yao and Zhu, we can see that many manufacturing methods provide a better stretch performance^28^. In fact, several techniques allow a strain higher than 200% and provide stable resistances to strains higher than 100%. However, most techniques with high strains have a significantly high initial resistivity, which limits their applications. Merilampi *et al.* reported similar sheet resistances, but the thickness of their samples was twice as high as ours, resulting in a two times higher resistivity value^31^.

**Figure 3:**
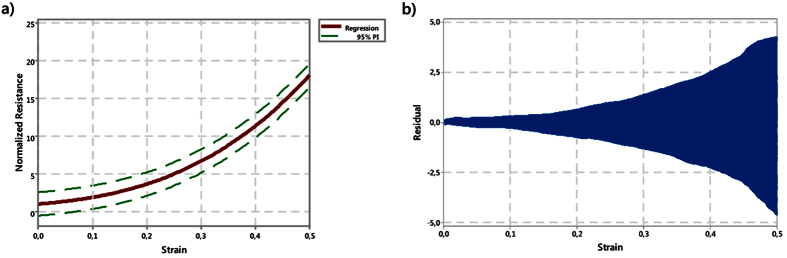
Regression model of the normalized resistance vs. strain performance of the stretchable interconnects. (**a**) The cubic regression model of strain performance and 95% Prediction Interval (PI) lines. The equation of the regression model is 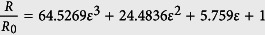. (**b**) Residuals of the regression model as a function of strain. Residual is the difference between observed value and fitted value.

Figure 2b shows that resistance grows exponentially in relation to strain, and that such growth was observed for all the 30 measured samples. Figure 2c shows that no sample broke before 50% strain and Fig. 2d that their variance and range were still reasonable up to 50% strain. Consequently, a cubic regression model valid up to 50% strain was developed for this study. The model describes the relation between normalized resistance and strain. The model is shown in Fig. 3a, and its equation is as follows:


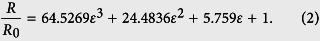


In equation 2, 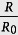 is the normalized resistance, and ε is the strain. For an estimation of the goodness-of-fit of the model, the residuals are shown in Fig. 3b as a function of strain. A residual is the difference between fitted value and observed value. As could be predicted from Fig. 2d, the higher the strain, the more inaccurate the model. The regression model is introduced for two reasons. First, the resistivity of the printed line is not constant since metal flakes move in polymer matrix during the strain. Therefore, theoretical models, e.g. given by than N. Lu *et al.*^32^, cannot be used to estimate the resistance. Secondly, the regression model is easy to implement into numerical simulators.

### Cycling strain test and time-dependent recovery

**Figure 4:**
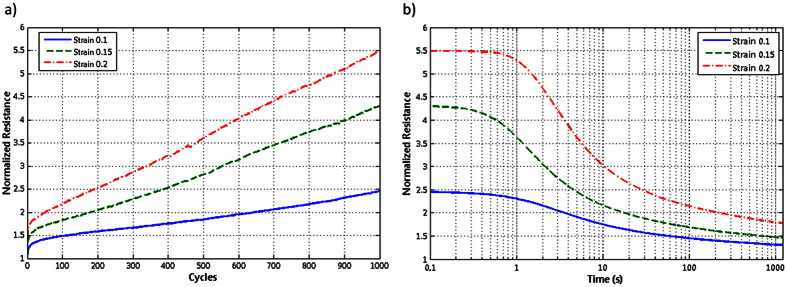
Normalized resistances of 10%, 15%, and 20% cycling tests. (**a**) Normalized resistances after each cycle up to 1000 cycles. (**b**) Normalized resistances after a 1000-cycle test as a function of time.

Cycling strain tests were performed to ensure the usability of the stretchable interconnects in textile applications. In the test, samples were first stretched 1000 cycles at a speed of 200 mm/min and then kept under constant 0% strain until their resistance values saturated. Cycling tests were performed for 10%, 15%, and 20% strains, and their results are shown in Fig. 4. Figure 4a shows normalized resistance as a function of cycle count. As can be seen, increasing as a function of cycles, the resistances measured 2.5, 4.3, and 5.3 times their initial resistances to 10%, 15%, and 20% strains, respectively. Figure 4b shows the measured normalized resistances to 10%, 15%, and 20% strains after cycling tests. Resistances decreased when strain was removed; i.e., their recovery was time-dependent. After one minute, the resistances decreased by more than half from 2.5, 4.3, and 5.3 to 1.5, 1.75, and 2.1, respectively. The normalized resistances to 10%, 15%, and 20% strains finally stabilized at 1.3, 1.4, and 1.7. These values are considerably lower than those initially measured in cyclic strain tests, which means that these test results are highly dependent on the timeframe in which resistances were measured. The time-dependent recovery was likely caused by the structural properties of TPU^33^.

The results in Fig. 4 show that resistance increases during cycling, but that it also recovers fast. Consequently, these screen-printed stretchable interconnects are suitable for many textile-integrated and epidermal applications. Comparison of the results in Figs 3b and 4b shows that the change in resistance due to plastic work is relatively small and thus expected to have minimal impact on the accuracy of the resistance model. The results presented in the next section attest the accuracy of the regression model given in equation (2) in prediction of the performance of a stretchable RFID tag under strain.

### Demonstration of a stretchable RFID tag

**Figure 5:**
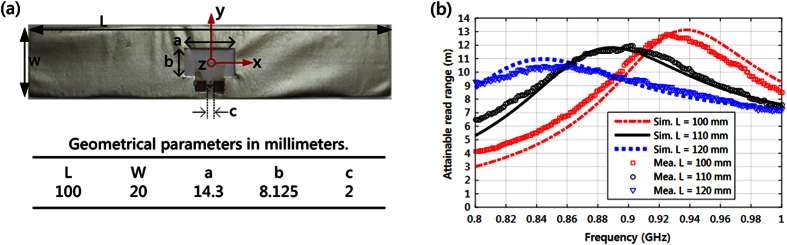
The screen-printed stretchable RFID tag. (**a**) The tag and the dimensions of the antenna shape. (**b**) Simulated and measured attainable read range. The reader antenna is located in the negative y-axis.

We screen-printed a dipole antenna on TPU to create a stretchable radio-frequency identification (RFID) tag. Because the antenna geometry differed from a narrow line, the printing parameters had to be adjusted for a high-quality pattern. Thus, the initial resistance test was repeated, and an initial sheet resistance of 50 mΩ/◽ was measured (fabricated tag shown in Fig. 5a). The RFID microchip fixture was mounted over the gap defined by parameter *c* in Fig. 5a. The pads of the microchip fixture were attached to the antenna with conductive epoxy. Since length is a decisive parameter for the electromagnetic properties of a dipole antenna, we chose to focus on the impact of the strain along the x-axis. Given the shape of the antenna, we also expected the percentage strains along the y-axis to be small in practice, but results from simulation where the strain is applied along y-axis are provided as supplementary information.

A full-wave electromagnetic solver (ANSYS HFSS), based on the finite element method, was used to model the antenna. In the simulation, we used the sheet resistance model given in equation 2 with an initial value of 50 mΩ/◽. As described in^7^, the simulated electromagnetic properties of the antenna together with the input impedance and wake-up power of the RFID microchip enabled estimation of the attainable read range of the tag (d_tag_), which was defined as the maximum distance at which the tag could be remotely powered in a perfectly anechoic environment under a given emission limit for the reader. In this work, we assumed the European RFID emission limit of 3.28 W of equivalent isotropically radiated power. In the measurement, we used the wireless method given in^34^. In the experiment, the tag was fixed to a block of foam at one end and measured in the non-stretched condition and then pulled to lengths of 11 (10% elongation) and 12 cm (20% elongation) (results shown in Fig. 4b). The downward shift in the frequency of peak d_tag_ is primarily due to the elongation of the antenna, which changes its input impedance and thus the frequency at which the microchip is complex-conjugate-matched with the antenna. The sheet resistance of the conductor increased with strain, causing a reduction in the peak value of d_tag_. Despite the frequency- and level-shifts, the tag maintained a high attainable read range of 9.5 m at 0.89 GHz. Importantly, the strain-dependent sheet resistance model could be utilized in antenna optimization in such a way that the maximum in the minimum d_tag_ over given elongations would occur at a desired frequency. Overall, the close agreement between simulated and measured results attests to the applicability of the developed strain-dependent sheet resistance model for high-frequency applications. The importance of our model is further highlighted by the data in the supplementary information section, which shows that converse results (increase in *d*_*tag*_ under strain) were obtained in simulations, where the initial sheet resistance of 50 mΩ/◽ was assigned also to the strained tag.

## Conclusions

This article deals with the manufacture of stretchable electronics by screen-printing from commercially available materials. Printed interconnects were characterized by measuring their initial electrical properties and electromechanical properties under strain. The manufactured interconnects measured a mean sheet resistance of 36.24 mΩ/◽, a value sufficient for most electronics applications. The interconnects easily withstood strains of 15-to−20%, which are within the range of those in textile-integrated and epidermal electronics applications. About half the samples withstood strains up to 74%. Because no sample broke before 50% strain, and because the variance in their strain performances was still low at this strain, a regression model valid up to 50% strain was developed to describe the normalized resistance vs. strain proportionality of the manufactured stretchable interconnects. Although the resistance of the conductors increased along with cyclic strains, the resistances were almost fully reversible, and this recovery was time-dependent and followed a logarithmic curve. Increases in permanent resistance were relatively small. In the end, we demonstrated the applicability of our strain-dependent sheet resistance model for high-frequency applications by simulating and measuring a stretchable RFID tag.

## Methods

### Screen-printing fabrication

All samples in our study were screen-printed on a 50-μm thick TPU substrate (Epurex Platilon U4201). Due to the softness of the substrate, sheets of it were attached tightly to 2-mm thick aluminum plates to secure a flat surface for printing. For conductive traces, a commercially available, highly conductive and highly flexible silver ink was used (CI-1036 by ECM). Before printing, the ink was pre-conditioned first by letting it warm up at room temperature for about an hour; then it was stirred by hand with a spatula for 2 minutes. Printing was done by using a TIC SCF-300 semi-automatic screen-printing machine. A polyester mesh screen was used in the printer. The mesh was attached to a 500 × 3000 mm^2^ aluminum frame with a profile of 30 × 30 mm^2^. The mesh count was 79 threads/cm, the thread diameter was 55 μm, and the mesh opening was 69 μm. The stretching angle of the mesh was 22.5°. The theoretical wet paste thickness with this screen was 26 μm. The hardness of the used squeegee was 75 Shore, and it was square-edged. The squeegee pressure was adjusted for the squeegee to bend about 45°. The ink deposition cycle was done two times for each pattern. After printing, the samples were oven-cured at 120° for half an hour.

### Initial electrical characterization

Electrical resistance was measured from the strain test samples using a Keithley 2425 sourcemeter and 2-point resistance measurement. Sheet resistance was calculated by dividing the resistance with the number of squares. The pattern in the strain test was 1 mm in width and 188.9 mm in length. Consequently, the number of squares in the pattern was 188.9.

### Electromechanical characterization

Stretchable samples were characterized by stretching them uniaxially and by measuring their electrical resistance in real time. For these tests, a custom test bench was designed and implemented. In the test setup, strain was exerted by fastening the ends of the sample to clamps attached to a linear actuator. One clamp on the linear actuator was static and the other moved. The initial distance between the clamps was 61 mm and the stretch rate 2.5 mm/min. The contact pads of the measured sample were positioned at the static end to reduce measurement noise. The actuator was powered by a Nanotec ST5709S1208 stepper motor. In one step, the motor rotated 0.90°. Because the screw lead of the actuator was 6 mm, the moving clamp traveled 6 millimeters when the motor rotated 360°. If one step of the motor was 0.90°, it took 400 steps for the moving clamp to travel 6 millimeters. Thus one motor step resulted in the moving clamp traveling 15 μm. The stepper motor was driven by an Arduino Uno R3 open-source electronic prototyping platform with an Arduino Motor Shield R3 attached. A supply voltage of 7 V was used to power the motor. Because the Arduino was programmed to provide a serial interface to control the linear actuator, the actuator could be controlled with a computer. Resistance was measured by using a Keithley 2425 sourcemeter, which was connected to the measured sample by placing needle probes on top of the pads of the sample. The sourcemeter, too, was connected to a computer. Testing was automated with LabVIEW software running on the computer. At each iteration of the loop in the LabVIEW software, the moving clamp was moved 15 μm, and the resistance was measured. When the target strain was reached, the software provided a data file with strain values as percentage values in one column and the corresponding resistance values in another column.

### Cyclic test

Cyclic tests were run using an Instron-4411 Universal Testing system. Samples were mounted on the system with similar clamps. Simultaneously, electrical parameters were measured with an iCraft AD-Converter Unit (model no. ADC ISO4x). The data was recorded with iPlotter software running on a laptop. The system had a 24-bit channel to measure voltage at a sampling frequency of 20 Hz. The unit had an input of 5 V and 400 mA with a 1-kΩ pull-up resistor. Resistance at all points was measured by post processing calculation. Samples were tested at three stretching rates: 10%, 15%, and 20%. Because the grip distance was 50 mm, the maximum strain was 5 mm, 7.5 mm, and 10 mm, respectively. The stretching speed was chosen as 200 mm/min. Samples were stretched 1000 cycles. After test cycles, resistance values were monitored until saturation.

### Characterization of the stretchable RFID tag

Printing parameters were adjusted for the antenna, thus the resistance measurement needed to be repeated. An initial value of 50 mΩ/◽ was measured. In addition, we measured the realized dimensions of the fabricated antenna (L = 101.6 mm, W = 19.8 mm, a = 14.6 mm b = 7.8 mm c = 1.9 mm) and used them to simulate the tag with a full-wave electromagnetic solver (ANSYS HFSS). The antenna conductor was modeled using an impedance boundary condition, which is a built-in feature in ANSYS HFSS. The input data for the boundary condition was the sheet resistance obtained from equation 2 for different strains. Based on^7^, dielectric constant and loss tangent of 3.2 and 0.1, respectively, were used to model the TPU substrate. The data we obtained from simulation was the input impedance and gain of the antenna as a function of frequency and antenna elongation. Since the studied dipole antenna remained less than half the wavelength in length for all studied strains, its radiation pattern was nearly uniform in the plane orthogonal to the dipole axis (yz-plane in Fig. 5a). Here, a slight difference compared with an ideal dipole which exhibits a perfectly uniform radiation pattern in this plane cut, was caused by the structural asymmetry introduced by the embedded impedance matching slot and gap for the microchip placement defined by parameters *a*, *b* and *c* in Fig. 5a. As a result, simulations showed that in yz-plane, *d*_*tag*_ was maximal along the negative y-axis and minimal along the positive y-axis for all studied frequencies and strains. The min/max difference was approximately 11% at the frequencies of the peak *d*_*tag*_. Thus, we chose the observation direction along negative y-axis for further analysis. In data processing, we used a wake-up power of −18 dBm (15.8 μW) and an equivalent circuit comprising a resistance of 2850 Ω connected in parallel to a capacitance of 0.91 pF to model the RFID microchip (NXP UCODE G2iL).

The measurement was conducted with an RFID tester (Voyantic Tagformance), which contained an RFID reader with an adjustable transmission frequency and output power. The tag was fixed at one end to a block of foam and measured in its non-stretched condition and then pulled to lengths of 11 and 12 cm. The foam had a very low permittivity and hence a minimal impact on the electromagnetic properties of the antenna. We recorded the lowest continuous-wave transmission power (threshold power: P_th_) at which the tag remained responsive in a frequency range of 800 MHz to 1 GHz. Here we defined P_th_ as the lowest power at which a valid 16-bit random number from the tag was received as a response to the query command in the ISO 18000-6 C communication standard. In addition, the wireless channel from the reader antenna to the location of the tested tag was characterized using a system reference tag with known properties. Based on the calibration data provided by the manufacturer of the measurement system, we estimate the combined static uncertainty for an attainable read range to be less than 5% -considering the variability in the system reference tag and the output power meter of the reader.

## Additional Information

**How to cite this article**: Suikkola, J. *et al.* Screen-Printing Fabrication and Characterization of Stretchable Electronics. *Sci. Rep.* **6**, 25784; doi: 10.1038/srep25784 (2016).

## Supplementary Material

Supplementary Information
