# Supplementary material for: Screen-Printing Fabrication and Characterization of Stretchable Electronics
Source: Sci Rep. 2016 May 13;6:25784. doi: 10.1038/srep25784 (PMC4865752; doi:10.1038/srep25784)
Supplement: Supplementary Information [file srep25784-s1.pdf]

Screen-Printing Fabrication and Characterization of Stretchable Electronics

Jari Suikkola, Toni Björninen, Mahmoud Mosallaei, Timo Kankkunen, Pekka Iso-Ketola, Leena Ukkonen, Jukka Vanhala, and Matti Mäntysalo*

Tampere University of Technology, Department of Electronics and Communications Engineering, Tampere, Korkeakoulunkatu 3, FI33720, Finland

* Corresponding author: matti.mantysalo@tut.fi

Supplementary information

In the article text, the impact of strain on the RFID tag was studied assuming the strain was applied along x-axis in Fig. 5. This coincides with the axis of the dipole antenna and thus the strain increases its electrical length. This has a major impact on the antenna input impedance. Another parameter affected by the strain is the length of the slot around the RFID microchip (parameter a in Fig. 5). The slot functions as an embedded impedance transformer which conjugate-matches the antenna impedance with the RFID microchip so that efficient power transfer is achieved between the two. Hence, the strain along x-axis impacts the operation of the tag through two different electromagnetic mechanisms. Strain along y-axis, on the other hand, affects the tag primarily through modification of the slot width (parameter b in Fig. 5) only. To provide further insight on the difference in the impact of strain along x- and y-axis, we studied both cases in simulations. The results in Fig. S1 show that overall, the strain along y-axis results in a smaller shift in frequency compared with strain along x-axis. The level shifts in both cases are approximately equal. For instance, the peak frequency of d_{tag} under 25% of strain along x-axis (W = 25 mm) coincides with that of only 10% strain along x-axis (L = 110 mm). Despite the frequency- and level-shifts, the tag maintained the high attainable read ranges of 9.5 m at 0.89 GHz and 10.1 m at 0.88 GHz and under 20% and 25% strains along x- and y-axis, respectively. Finally, we corroborated the important role of the strain-dependent sheet resistance model in this application through another simulation where the sheet resistance of 50 mΩ/□ measured from the unstrained antenna was assigned also to the antenna under strain. As seen from Fig. S2a, the simulation predicts that strain would increase d_{tag}. This, however, is in a clear contradiction with the measured data presented in Fig. S2.

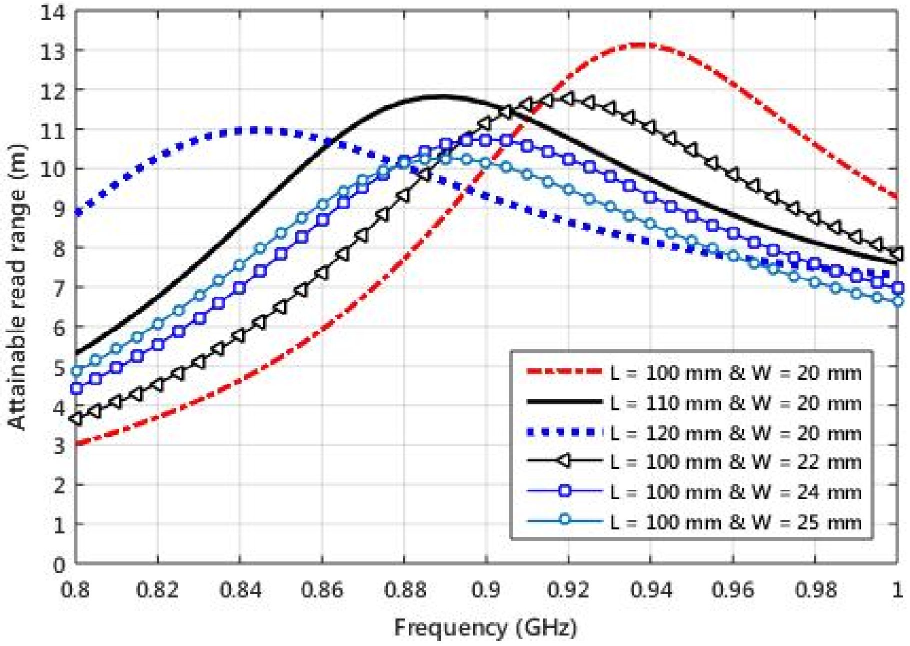

Fig. S1. Simulated attainable read range of the tag under strain along x- or y-axis. . The reader antenna is located in the negative y-axis in Fig. 5.

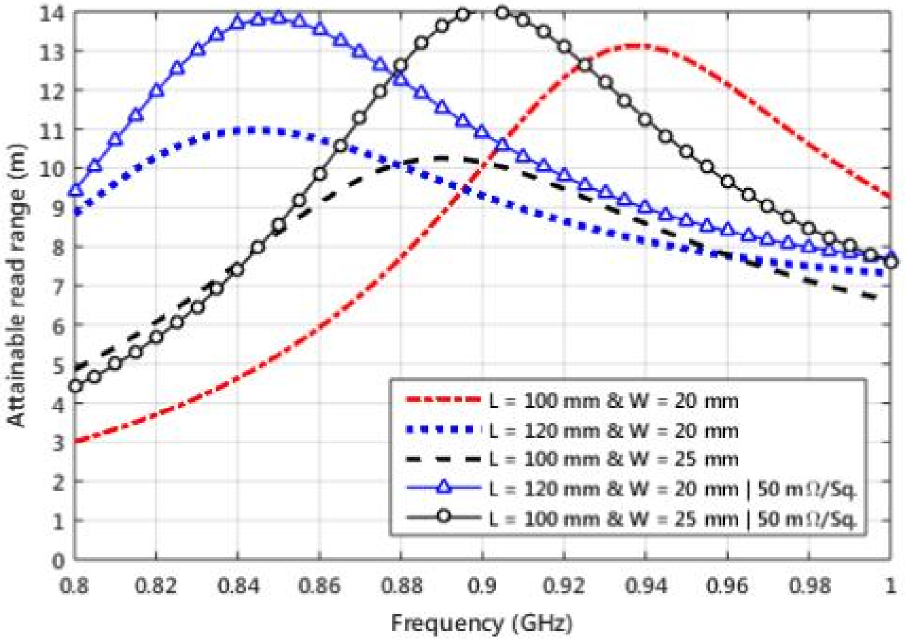

Fig. S2. Simulated attainable read range of the tag under strain along x- or y-axis and either strain-dependent or constant sheet resistance assigned on the antenna. . The reader antenna is located in the negative y-axis in Fig. 5.
